# Hypertrophic adenoids in patients with nasopharyngeal carcinoma: appearance at magnetic resonance imaging before and after treatment

**DOI:** 10.1186/s40880-015-0005-y

**Published:** 2015-03-05

**Authors:** Yao-Pan Wu, Pei-Qiang Cai, Li Tian, Jie-Hua Xu, Richard Alan Mitteer, Yi Fan, Zhenfeng Zhang

**Affiliations:** State Key Laboratory of Oncology in South China, Collaborative Innovation Center for Cancer Medicine, Guangzhou, Guangdong 510060 P. R. China; Center of Medical Imaging & Image-guided Therapy, Sun Yat-sen University Cancer Center, 651 Dongfeng Road East, Guangzhou, Guangdong 510060 P. R. China; Department of Nuclear Medicine, The Third Affiliated Hospital of Sun Yat-sen University, Guangzhou, Guangdong 510630 P. R. China; Department of Radiation Oncology, Perelman School of Medicine, University of Pennsylvania, 3400 Civic Center Blvd, Philadelphia, PA 19104 USA

**Keywords:** Hypertrophic adenoids, Nasopharyngeal carcinoma, Magnetic resonance imaging

## Abstract

**Introduction:**

Patients with nasopharyngeal carcinoma (NPC) sporadically develop abnormal adenoids. Nasopharyngeal adenoids are usually included in the gross tumor volume (GTV) but may have different therapeutic responses than tumor tissue. Therefore, distinguishing adenoids from tumor tissue may be required for precise and efficient chemoradiotherapy and radiotherapy. We characterized nasopharyngeal adenoids and investigated the therapeutic responses of NPC and nasopharyngeal adenoids using magnetic resonance imaging (MRI).

**Methods:**

MRI data from 40 NPC patients with a coexisting adenoid mass before and after treatment were analyzed. The features of the adenoid masses, including location, striped appearance, size, interface, symmetry/asymmetry, and cysts, were evaluated. Treatment response were scored according to the World Health Organization guidelines.

**Results:**

A striped appearance was observed in 36 cases before treatment and in all cases after treatment. In these 36 cases, the average GTVs including and excluding the uninvolved adenoids were 19.8 cm^3^ and 14.8 cm^3^, respectively. The average percentage change after excluding the uninvolved adenoids from the GTV was 31.0%. Stable disease in the adenoids was identified in 27 (96.4%) of 28 patients after neoadjuvant chemotherapy, while NPC clearly regressed. Partial adenoid responses were identified in 33 (82.5%) of 40 patients at 3 months after chemoradiotherapy or radiotherapy, whereas complete tumor responses were achieved in all patients. Six months after treatment, the adenoids continued to atrophy but did not disappear, and tumor recurrence was not found.

**Conclusions:**

Nasopharyngeal adenoids and carcinoma tissue in NPC patients can be distinguished by using MRI and have different responses to chemoradiotherapy and radiotherapy. These findings contribute to better delineating the GTV of NPC, based on which spatially optimized strategies can be developed to render precise and efficient chemoradiotherapy and radiotherapy. Additionally, we observed a clear difference in the responses of these two tissue types to current therapies. This finding may reduce or avoid unnecessary biopsies or overtreatment.

## Background

Nasopharyngeal carcinoma (NPC) is the most common cancer that originates in the nasopharynx. The nasopharyngeal adenoids, which are part of the Waldeyer’s ring, enlarge and reach a maximum size in children of 3 to 7 years old, after which these tissues usually atrophy with aging [1,2]. The adenoids may persist or recur for several reasons, including infections and irritants [3]. In South China, where NPC is an epidemic disease [4], the coexistence of hypertrophic adenoids and NPC is not rare, particularly in young people. In the current clinical practice of radiotherapy, hypertrophic adenoids are considered part of the gross tumor volume (GTV), which serves as a key determinant for radiation dosing. However, GTV should not include the hypertrophic adenoids, according to its definition. The imaging distinction of hypertrophic adenoids from adjacent tumor tissue will allow the precise measurement of GTV to avoid overdosed radiation and guide the spatial delivery of radiation to reduce non-specific toxicity in normal tissue. Furthermore, the adenoids and carcinoma are unique tissues that may have different responses to chemoradiotherapy and radiotherapy. Better understanding the difference between these tissues by using non-invasive imaging approaches will contribute to optimizing the therapeutic dose and eliminating unnecessary biopsies.

Currently, magnetic resonance imaging (MRI) is the best modality for assessing NPC. MRI examination on the nasopharynx offers an accuracy of 95% for detecting NPC and can discriminate benign disease from NPC with an overall specificity of 93% [5]. This highly accurate imaging influenced the hypothesis that NPC and hypertrophic adenoids could be differentiated by using MRI before treatment. In the present study, we analyzed the appearances of hypertrophic adenoids and NPC before and after treatment by using MRI with a focus on T1-weighted contrast-enhanced imaging. We also characterized hypertrophic adenoids and their treatment responses in NPC patients.

## Methods

### Patient selection

The institutional review board of Sun Yat-sen University Cancer Center has approved this retrospective study in compliance with the Helsinki Declaration.

Patients with NPC coexisting with hypertrophic adenoids were identified in the pathology databases at Sun Yat-sen University Cancer Center between January 2007 and January 2011. The selection criteria were as follows: 1) MRI was performed before and after treatment; 2) patients had a nasopharyngeal adenoid hypertrophy greater than 10 mm (measured at the greatest anteroposterior diameter on axial T1-weighted contrast-enhanced images before treatment); and 3) the diagnosis of hypertrophic adenoids was confirmed in biopsy specimens.

### T staging and subtype classification of NPC

NPC T staging was performed according to the newly released 7th edition of the Union for International Cancer Control (UICC) staging system. The World Health Organization (WHO) recognizes three subtypes of NPC: type 1, squamous cell carcinoma; type 2, non-keratinizing carcinoma; and type 3, undifferentiated carcinoma.

### Treatment

The treatment for childhood and adolescent patients with NPC followed the guidelines that were established for adults. For patients who received neoadjuvant chemotherapy followed by chemoradiotherapy or radiotherapy, follow-up MRI studies were performed approximately 10 days after neoadjuvant chemotherapy completion and 3 and 6 months after chemoradiotherapy or radiotherapy completion. For patients who received chemoradiotherapy or radiotherapy without neoadjuvant chemotherapy, follow-up MRI studies were performed at 3 and 6 months after treatment completion.

### MRI protocol

MRI examinations were performed by using a 1.5 T MRI scanner (Signa CV/i, GE Healthcare, Milwaukee, WI, USA) or a 3 T MRI scanner (Trio Tim, Siemens Medical Systems, Erlangen, Germany) with a head and neck combined coil. T2-weighted images in the axial planes and T1-weighted images in the axial, sagittal, and coronal planes were obtained before the injection of contrast material. After the intravenous injection of gadopentetate dimeglumine at a dose of 0.1 mmol per kg body weight, T1-weighted axial, sagittal, and coronal sequences with fat saturation were performed sequentially using parameters similar to those for pre-injection imaging. The section thicknesses and intersection gaps in the axial plane were 4 mm and 1 mm, respectively.

### MRI evaluation

Two radiologists (with 10 and 5 years of experience, respectively, in head and neck imaging) evaluated the images of nasopharyngeal lesions using a Picture Archiving and Communication System (Centricity Radiology RA1000, GE Medical Systems Integrated Imaging Solutions, Mount Prospect, IL, USA). Consensus interpretations were accepted in cases with discrepancies.

For the purpose of this study, the roof and the posterior superior nasopharyngeal wall were defined on axial MRI sections as being above and level, respectively, with the distal ends of the torus tubarius [6].

The MRI features of hypertrophic adenoids were recorded, including location, striped appearance, size, the interface between hypertrophic adenoids and NPC, symmetry/asymmetry, cyst formation, and the presence of hypertrophic adenoids that extend into the pharyngeal recesses. A normal striped appearance consists of alternating dark and bright vertical stripes of hypo- and hyper-enhanced tissues [5,7]. This striped appearance was also recorded after treatment as some of the stripes may appear only after treatment because of tumor involvement. The greatest anteroposterior diameter of hypertrophic adenoids was measured based on the visualization of stripes before treatment; if the stripes only appeared after treatment, then the greatest anteroposterior diameter was measured after treatment. The interface between hypertrophic adenoids and NPC was classified as well-defined or ill-defined. Cyst formation was defined by the presence of an area that is hypo-intense on T1-weighted MR images obtained before and after enhancement and that is markedly hyper-intense on T2-weighted MR images. When hypertrophic adenoids are markedly large, they may extend into the pharyngeal recesses (hypo-enhanced band similar to the dark stripes in the hypertrophic adenoids) [6]. The presence of hypertrophic adenoid extension into the pharyngeal recesses was recorded.

Familiarity with the MRI appearances of hypertrophic adenoids (if still in existence) after treatment is necessary to reduce overtreatment and unnecessary biopsies. Therefore, the characteristics of hypertrophic adenoids after treatment were also reviewed.

The intact stripe closest to the interface was used as a safe margin between the tumor tissue and the uninvolved adenoids to improve GTV delineation. Before treatment, when the tumor tissue could be distinguished from the adenoids, the GTVs, both including and excluding the uninvolved adenoids, were calculated at the post-processing MRI workstation (GE SUN ADW 4.4) using a volume rendering application. Additionally, the percentage change at which the uninvolved adenoids could be excluded from the GTV was also calculated. The location of this safe stripe was roughly classified as being in 1 of 3 sites. Site 1 was near the pharyngeal recess ipsilateral to the site of primary tumor (supposing that the NPC was located at the pharyngeal recess or nasopharyngeal lateral wall). In this case, all hypertrophic adenoids were present and remained uninvolved. Site 2 was between site 1 and the midline of the nasopharynx. In this case, approximately half of the hypertrophic adenoid volumes were left. Site 3 was between the midline of the nasopharynx and the pharyngeal recess contralateral to the site of primary tumor. In this case, only a few hypertrophic adenoids were present. When the NPC originated in the posterior superior nasopharyngeal wall and/or the roof where hypertrophic adenoids normally reside, no safe margin existed between the two tissues. This case was classified as site 3. The frequencies of these three sites were recorded.

According to the new Response Evaluation Criteria in Solid Tumors Guideline (version 1.1) from 2009 [8], the response criteria were defined as follows: complete response (CR), no evidence of disease; partial response (PR), a ≥ 30% decrease in the sum of the greatest dimensions of the target lesions and no evidence of new lesions or progression of any lesion; progressive disease (PD), a ≥ 20% increase in the sum of the greatest diameters of the target lesions or the appearance of new lesions; and stable disease (SD), small changes that did not meet the criteria for PR or PD. In addition to these criteria, a near-CR was added and defined as an extremely good PR with little visual disease left [9].

## Results

### The clinical characteristics of patients

Forty patients fit the criteria and were eligible for this retrospective study: 30 were childhood and adolescent patients (younger than 18 years), and 10 were adult patients (greater than or equal to 18 years), with a median age of 16 years (range, 9–51 years); 31 were males (median age, 16 years; range, 9–40 years), and 9 were females (median age, 15 years; range, 11–51 years). Among the 40 patients, 1, 6, 21, and 12 had T1, T2, T3, and T4 NPC, respectively. All patients were pathologically diagnosed with type 3 disease.

### MRI appearances of hypertrophic adenoids before and after treatment

**Figure 1:**
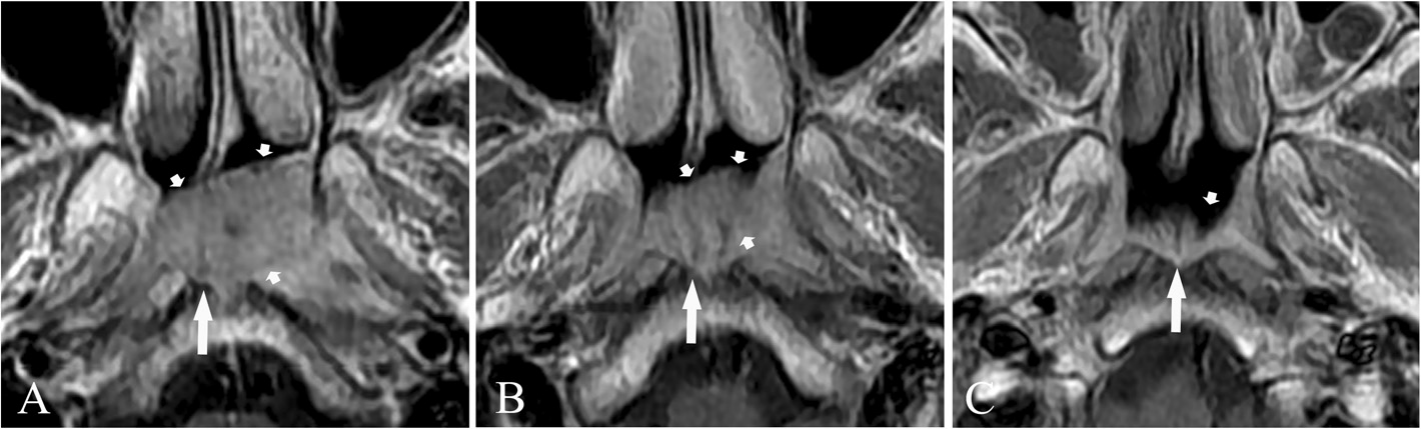
**Treatment response, as determined by axial T1-weighted contrast-enhanced magnetic resonance imaging (MRI), in a 12-year-old nasopharyngeal carcinoma (NPC) patient with a small tumor coexisting with hypertrophic adenoids. A**, before treatment, the NPC originated in the roof of the nasopharynx (short arrows) and could not be clearly distinguished from hypertrophic adenoids (long arrow). **B**, 10 days after the end of neoadjuvant chemotherapy, a partial response (PR) was achieved for the tumor (short arrows), and stripes (long arrow) became visible in hypertrophic adenoids. **C**, 3 months after the end of radiotherapy, a complete response (CR) was achieved for the tumor, a PR was demonstrated for hypertrophic adenoids, and the stripes on hypertrophic adenoids became more visible (long arrow). Note that hypertrophic adenoids were asymmetric (short arrow) most likely because of previous tumor invasion.

**Figure 2:**
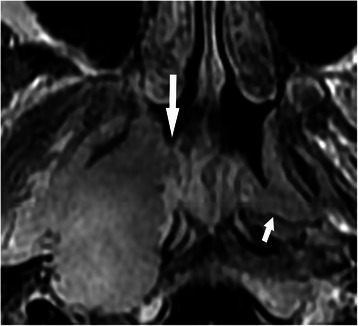
**Axial T1-weighted contrast-enhanced MRI before treatment of a 13-year-old boy who had NPC coexisting with hypertrophic adenoids.** The interfaces between NPC and hypertrophic adenoids are ill-defined, indicating that the adenoids were invaded (long arrow). The presence of hypertrophic adenoid extension into the left pharyngeal recesses is also detected (short arrow).

**Figure 3:**
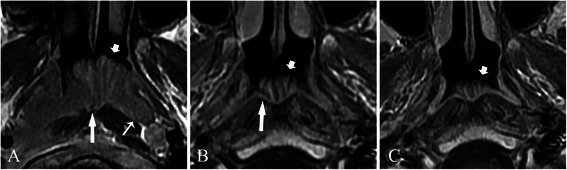
**Treatment response, as determined by axial T1-weighted contrast-enhanced MRI, in a 12-year-old patient with NPC coexisting with hypertrophic adenoids. A**, before treatment, hypertrophic adenoids (short arrow) were invaded by the tumor, which originated from the right pharyngeal recess. The bright stripe in hypertrophic adenoids was interrupted (long arrow). Hypertrophic adenoid extension into the left pharyngeal recesses is also detected (thin long arrow). **B**, 3 months after the end of chemoradiotherapy, a CR was achieved for the tumor, and a PR was demonstrated for hypertrophic adenoids (short arrow). Note that the previously invaded stripes retained their hypo-intensity (long arrow). **C**, 6 months after the end of chemoradiotherapy (without further treatment), no tumor recurrence was identified, and hypertrophic adenoids became smaller (short arrow).

**Figure 4:**
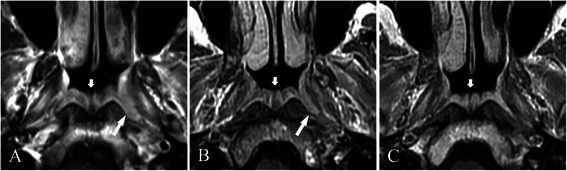
**Treatment response, as determined by axial T1-weighted contrast-enhanced MRI, in a 40-year-old male NPC patient with a small tumor coexisting with hypertrophic adenoids. A**, before treatment, a small tumor was confined to the mucosa of the left pharyngeal recess (long arrow), and hypertrophic adenoids were located in the posterior superior and roof of the nasopharyngeal wall (short arrow). **B**, 10 days after the end of neoadjuvant chemotherapy, a PR was achieved for the tumor (long arrow). **C**, 3 months after the end of chemoradiotherapy, a CR was achieved for the tumor. Stable disease (SD) is demonstrated for hypertrophic adenoids after treatment (short arrows in **B** and **C**).

**Figure 5:**
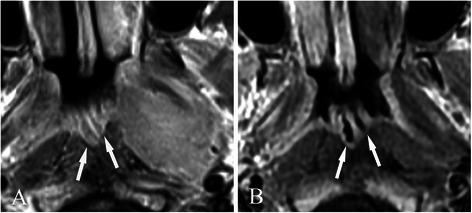
**Axial T1-weighted contrast-enhanced MR images of a 16-year-old child who had NPC coexisting with hypertrophic adenoids. A**, before treatment, dark stripes are observed in hypertrophic adenoids (arrows). **B**, 3 months after radiotherapy, the stripes disappear and are replaced by air (arrows).

All hypertrophic adenoids were located in the posterior superior nasopharyngeal wall and roof. Stripes could not be detected in 4 patients due to complete involvement by the tumor (Figure 1A), but were demonstrated in 36 patients before treatment (Figures 1B, C, 2, 3, 4 and 5). The mean greatest anteroposterior diameter of hypertrophic adenoids was 16 mm (range, 10–22 mm). The interface between hypertrophic adenoids and NPC was well-defined in 5 patients and ill-defined in 35 patients (Figure 2). Only 5 patients had hypertrophic adenoids with a symmetrical appearance. Nasopharyngeal cysts in hypertrophic adenoids were identified in 25 patients (62.5%). Thornwaldt cysts, which were identified by their midline location in the nasopharyngeal roof, were present in 2 patients (5.0%). Hypertrophic adenoids extending into the pharyngeal recess (Figures 2 and 3A) were detected in 26 patients (65.0%).

At 3 and 6 months after the end of treatment, stripes were demonstrated in all patients. In addition to atrophy, other characteristics of hypertrophic adenoids, such as asymmetry (Figure 1C) and air in the dark stripes (Figure 5B), were also observed in 18 (45.0%) and 7 (17.5%) patients, respectively.

### The volume, percentage change, and safe margin sites

The tumor tissue could be distinguished from the adenoids in 36 patients before treatment. In these patients, the average GTVs including and excluding the uninvolved adenoids were 19.8 cm^3^ and 14.8 cm^3^, respectively. Additionally, the average percentage change in which the uninvolved adenoids could be excluded from the GTV was 31.0%.

Safe margins at sites 1 (Figure 4A), 2 (Figures 2 and 3), and 3 were identified in 5 (12.5%), 17 (42.5%), and 18 (45.0%) patients, respectively. Only 1 case of NPC originated in the roof of the nasopharynx (Figure 1A), whereas other cases primarily originated in the pharyngeal recess.

### Treatment response

The MRI-determined treatment responses (Figures 1, 3, and 4) are provided in Table 1. A CR for the tumor was achieved in all patients at 3 months after the end of chemoradiotherapy or radiotherapy. Hypertrophic adenoids were atrophied and did not disappear at 3 or 6 months after treatment. At 6 months after the end of treatment, no tumor recurrence was found.

**Table 1:** **Treatment response, as determined by magnetic resonance imaging (MRI), during and after treatment in patients with nasopharyngeal carcinoma (NPC) and hypertrophic adenoids**

**Assessment point**	**No. of patients**	**Hypertrophic adenoid response**	**NPC response**
Approximately 10 days after the end of neoadjuvant chemotherapy	28	SD, 27; PR, 1	PR, 18; near-CR, 6; CR, 4
Three months after the end of CRT or RT			
Patients who did not receive neoadjuvant chemotherapy	12	PR, 12	CR, 12
All patients	40	PR, 33; near-CR, 7	CR, 40
Six months after the end of CRT or RT (all patients)	40	PR, 13; near-CR, 27	CR, 40

SD, stable disease; PR, partial response; CR, complete response; near-CR, very good partial response; CRT, chemoradiotherapy; RT, radiotherapy.

## Discussion

In this study, we calculated the GTVs including and excluding the uninvolved adenoids and the percentage change at which the uninvolved adenoids could be excluded from the GTV. Additionally, we analyzed the MRI appearances of hypertrophic adenoids before and after treatment and the treatment responses of NPC and hypertrophic adenoids. The results of our study demonstrated that the tumor tissue and the uninvolved adenoids could be distinguished on MRI by the safe margin before treatment in 90.0% of all patients. More importantly, the average change at which the uninvolved adenoids could be excluded from the GTV was 31.0%. These results allowed better GTV delineation and a considerable reduction in the radiation dose delivered to the normal adjacent tissues. Another finding was that the tumors disappeared at 3 months after treatment, whereas hypertrophic adenoids remained with varying degrees of atrophy in all patients, as determined by MRI. Different treatment responses of NPC and hypertrophic adenoids were clearly detected.

The current clinical practice of radiotherapy includes hypertrophic adenoids as part of the GTV. However, when the tumor is small, particularly for those with safe margins at site 1, the radiation dose delivered to hypertrophic adenoids may be several times larger than that of the tumor tissue. Meanwhile, due to the age-associated changes in hypertrophic adenoids, they may more frequently coexist in childhood and adolescent NPC patients than in adult NPC patients. Childhood and adolescent patients are at a higher risk of long-term complications after treatment. Thus, distinguishing these tissues is critical for reducing the radiation dose delivered to the adjacent normal tissues, particularly in children and adolescents. Fortunately, in 90.0% of all patients, the tumor tissue and uninvolved adenoids could be distinguished by MRI before treatment, and the average volume of uninvolved adenoids was considerable.

Several factors contribute to the finding that these two tissues could be distinguished by MRI. First, hypertrophic adenoids have a characteristic stripe appearance on MR images. This stripe appearance is a distinguishing feature of the benign enlargement of hypertrophic adenoids [5]. Second, a high percentage of hypertrophic adenoids extending into the pharyngeal recesses and nasopharyngeal cysts was detected, which supports the diagnosis of hypertrophic adenoids. Third, the common sites for these two tissues are different, which provides a greater opportunity to distinguish NPC and hypertrophic adenoids. The pharyngeal recess is the most common site for early NPC [10], whereas hypertrophic adenoids are always located in the posterior superior nasopharyngeal wall and roof. Lastly, because NPC and hypertrophic adenoids are different tissues, appreciable differences in their signal intensity can be observed.

In South China, hypertrophic adenoids are uncommon in patients with NPC but not rare. In our study, we only included those hypertrophic adenoids with a greatest anteroposterior diameter larger than 10 mm because larger radiation doses are delivered to patients with larger hypertrophic adenoids according to the current treatment strategies for NPC. We also artificially classified three sites for the safe margins between NPC and uninvolved adenoids. Clearly, the more uninvolved the adenoids are (such as those with safe margins in sites 1 and 2), the more the radiation dose is reduced for hypertrophic adenoids.

In our work, we closely examined the involved stripes. When the bright stripe of hypertrophic adenoids was involved, the stripe became dark and was interrupted. Thus, this stripe was easier to detect than if the involved stripe was dark (Figure 3A and B) because the difference in signal intensity between the bright stripe and the tumor were more obvious than that between the dark stripe and the tumor. Thus, we suggest that the continuity of the bright stripe closest to NPC should be used as a reliable boundary to indicate the involved and uninvolved parts of hypertrophic adenoids.

In this study, we demonstrated different treatment responses between NPC and hypertrophic adenoids. Most hypertrophic adenoids underwent little to no change, whereas the tumor tissues clearly regressed after neoadjuvant chemotherapy. Three months after the end of chemoradiotherapy or radiotherapy, a PR for hypertrophic adenoids was achieved in 77.1% of the patients, whereas a CR for the tumor tissues was achieved in all patients. In summary, NPC regressed faster than hypertrophic adenoids due to their tissue characteristics. Familiarity with the different treatment responses of these two tissues on MRI may reduce or avoid unnecessary biopsies or overtreatment.

Our study has one obvious limitation. Although our results demonstrated that excluding the uninvolved adenoids from GTV delineation would be a feasible way to improve the efficacy of radiotherapy on NPC, we did not have clinical data to validate this hypothesis because current GTV delineation still covers adenoids in clinical practice. Our hypothesis needs to be verified in future studies.

## Conclusion

Hypertrophic adenoids and carcinoma tissues in NPC patients can be distinguished by MRI and have different responses to chemoradiotherapy and radiotherapy. These findings contribute to better defining the GTV of NPC based on which spatially optimized strategies can be developed to render precise and efficient chemoradiotherapy and radiotherapy. The latter finding also contributes to reducing or avoiding unnecessary biopsies or overtreatment.

## References

[1] Fujiyoshi T, Watanabe T, Ichimiya I, Mogi G (1989). Functional architecture of the nasopharyngeal tonsil. Am J Otolaryngol.

[2] Vogler RC, Ii FJ, Pilgram TK (2000). Age-specific size of the normal adenoid pad on magnetic resonance imaging. Clin Otolaryngol Allied Sci.

[3] Wang MC, Tsai TL, Liu CY, Shu CH, Lin CZ (2003). Nasopharyngeal lymphoid hyperplasia of an HIV carrier, mimicking nasopharyngeal cancer. J Chin Med Assoc.

[4] Wei KR, Zheng RS, Zhang SW, Liang ZH, Ou ZX, Chen WQ (2014). Nasopharyngeal carcinoma incidence and mortality in China in 2010. Chin J Cancer.

[5] King AD, Vlantis AC, Bhatia KS, Zee BC, Woo JK, Tse GM (2011). Primary nasopharyngeal carcinoma: diagnostic accuracy of MR imaging versus that of endoscopy and endoscopic biopsy. Radiology.

[6] Bhatia KS, King AD, Vlantis AC, Ahuja AT, Tse GM (2012). Nasopharyngeal mucosa and adenoids: appearance at MR imaging. Radiology.

[7] King AD, Vlantis AC, Tsang RK, Gary TM, Au AK, Chan CY (2006). Magnetic resonance imaging for the detection of nasopharyngeal carcinoma. AJNR Am J Neuroradiol.

[8] Eisenhauer EA, Therasse P, Bogaerts J, Schwartz LH, Sargent D, Ford R (2009). New response evaluation criteria in solid tumours: revised RECIST guideline (version 1.1). Eur J Cancer.

[9] Buehrlen M, Zwaan CM, Granzen B, Lassay L, Deutz P, Vorwerk P (2012). Multimodal treatment, including interferon beta, of nasopharyngeal carcinoma in children and young adults: preliminary results from the prospective, multicenter study NPC-2003-GPOH/DCOG. Cancer.

[10] Wei WI, Sham JS, Zong YS, Choy D, Ng MH (1991). The efficacy of fiberoptic endoscopic examination and biopsy in the detection of early nasopharyngeal carcinoma. Cancer.

